# Notes and Notices

**Published:** 1898-09

**Authors:** 


### NOTES AND NOTICES.

S. G. MacCracken, M. D., has removed from Winnetka to Elgin, Ill. Residence, 279 Douglas Avenue, Spurling Block.

